# Medico-Chirurgical Transactions. Vol. XXVIII

**Published:** 1846-01

**Authors:** 


Medico-chirurgical Transactions, published by the Royal Medical and Chirurgical Society of London. Volume the Twenty-eighth (the Tenth of the Second Series). Longman and Co. 1845.

In presenting to the notice of our readers another volume of the Transactions of the Royal Medical and Chirurgical Society, we have much pleasure in congratulating the Fellows on the successful progress of the Society, which is fortunate in having elected as President, a gentleman of the highest character as an accomplished physician and practitioner. During a season in which the elements of disorder were rife in every department of the profession—in which angry feelings were engendered by the collision of opposing interests and different views in relation to medical politics, all parties met as friends in the room of the Medico-chirurgical Society, to further the objects of medical science, and to communicate and interchange their views of practice. The more important points of the labours of the Session are exhibited in the volume before us, which contains a series of papers of great value and interest. We are happy to learn that the improved state of the finances of the Society has led the Council to act with increased liberality towards the contributors to the Transactions. The authors are not now obliged to bear any portion of the expense of the plates illustrating their papers—not even of the coloring of them, and they receive a limited number of private copies free of all cost. The present volume, though containing a less number of papers than its predecessor, forms a bulky book of 623 pages, owing to the great length of some of the communications; indeed, one of the papers occupies so much space that it might well have been published in an independent form.

I. Case in which the Vena Cava Inferior was obstructed from the commencement of the Common Iliac Veins, and its Cavity entirely obliterated between the Entrance of the Emulgent and Hepatic Veins. By *Thomas B. Peacock*, M.D. Physician to the Royal General Dispensary, Aldersgate Street.

The author remarks that, though a considerable number of instances are recorded, in which the main venous trunks of the upper or lower portions of the body have been found more or less completely obstructed, it is conceived that the following case, which furnishes an unusual example of an entire obliteration of the inferior cava, will be interesting to the Society.

Agnes M'Ewen, aged 47, of intemperate habits, was admitted into the Royal Infirmary of Edinburgh, on the 16th of August, 1842; she had been married at the age of 25; had had six children, and always recovered well after her confinements. In the October preceding, her health began to decline, and the catamenia appeared more frequently, and the discharge was more copious than previously. "In the course of the spring she received a severe blow on the abdomen, and the following day had hæmorrhage from the bowels. After this her health rapidly gave way, and the catamenia ceased; the abdomen became swollen, and one month before her admission her legs also were observed to be œdematous, the urine being at the same time scanty and high-coloured. Two days before her death she was seized with profuse vomiting of blood, and when received into the Infirmary was greatly prostrated, her countenance was pale, puffy, and sallow, the limbs very œdematous, and the abdomen large and tense. Shortly after being removed to the ward, the hæmatemesis recurred, and bloody fluid was discharged from the bowels. She sank eight hours after her admission."

Autopsy 37 hours after death. The following were the more important morbid appearances. Body was anasarcous and abdominal cavity contained ten pints of a pale straw-coloured fluid. Old adhesions on both sides of the chest; on the left side, the false membrane was fibro-cartilaginous and full half-an-inch in thickness, and the lung was of small size, and the corresponding portion of the chest contracted. The stomach contained a large quantity of thin bloody fluid, but its mucous membrane was healthy. The liver was extremely small, weighing only 24 ozs., but free from disease. The left kidney was reduced to a very small size, weighing only $1\frac{1}{2}$ oz. The pelvis was expanded, and the striated portion almost entirely destroyed. The right kidney was large, weighing $5\frac{1}{2}$ ozs.—it was pale and granular. The uterus was somewhat larger than usual and attached by firm adhesions to the rectum, sigmoid flexure of the colon, and the sides of the pelvic cavity.

"On removing this organ from the body, the veins in its substance, in the broad ligaments, and around the ovaries, were found distended by firm fibrinous clots, and on following the venous branches, in the course of the circulation, the common iliac veins displayed similar coagula, and they thence extended continuously throughout the cava, till, about $1\frac{1}{2}$ in. below its passage through the diaphragm, that vessel became reduced to an impervious ligamentous cord. The fibrinous clots on the right side, though firmly obstructing the common iliac vein, ceased immediately above its division; but on the left side they extended both into the hypogastric and external iliac veins, entirely closing their cavities. The left common iliac vein was firmly adherent to its corresponding artery.

"In the veins of the uterus and ovaries, the coagula had a peculiar fusiform shape, and were firmly adherent to the coats of the vessels, generally by one extremity: they were very solid, so large as to distend the vessels, and generally about half an inch in length, and were composed of lymph in layers of a pale colour, alternating with others of a pinkish hue. In the iliac vessels and cava they consisted of continuous masses, distinctly laminated, not unlike the fibrinous layers of an aneurismal sac. Their colour in the iliacs and lower part of the cava was pale red, and they gradually became more decolorized as they advanced towards the heart; they were closely united to the tunics of the veins, and entirely filled their cavities. The spermatic veins were of large size, and their coats thicker and firmer than usual; the orifice of the left vein into the trunk of the emulgent, and of the right into the cava, were closed by firm fibrinous masses, and the course of the right spermatic vein was occupied by small fusiform clots adherent to its coats, and similar to those of the uterine veins.

"The calibre of the iliac veins, and especially of the cava, was much reduced, and their coats were indurated and thickened: this became more conspicuous towards the upper part of the vessel;—the cavity occupied by the clots gradually diminishing, and the coats increasing in thickness, till, as before stated, about an inch and a half below the passage of the vessel through the diaphragm, it became entirely obliterated, and was reduced to a mere cord, about the size of the little finger, of a glistening white colour, and firm fibrous texture.

"The communication of the left emulgent vein with the cava was open, and this vessel was throughout free from coagula; a vein of considerable size, also unobstructed, entered it from above. The right emulgent vein was closed by a large and closely adherent coagulum, projecting into its orifice." P. 5.

The heart, with the liver and other abdominal viscera, having been removed from the body before the obstruction of the cava was detected, the author was unable to institute so satisfactory an examination as would have been desirable, into the course by which the venous blood of the lower extremities had been returned to the heart. "On examination, however, the vena azygos was found of unusually large size, being immediately above the entrance of the vena azygos minor, fully one inch in circumference; the branches entering the right vein, and the vena azygos minor with its branches, and especially some from the spinal canal and intercostal spaces, were greatly enlarged. The portal trunk and branches were free from disease; the hepatic veins entered the only portion of the vena cava inferior which remained pervious." The veins of the integuments of the abdomen and those following the course of the internal epigastric artery, did not appear larger than usual.

The author remarks that the above case possesses several points of interest. "It evinces the facility with which the venous circulation can be maintained notwithstanding the entire obstruction of one of its main trunks. In this respect, it is not less remarkable than the case related by Dr. Baillie, in which 'the inferior cava was changed into a ligamentous substance, from the entrance of the emulgent veins to the right auricle of the heart,' yet we are assured that this condition was in no degree accessory to the death of the patient. Cases of this description, in which the obliteration of the vessels is complete and of long duration, are decidedly rare, though not a few instances are recorded of partial or recent obstructions in the cavities of the inferior and also of the superior cava."

The history of the case is unfortunately very defective, and leaves us in doubt whether the dropsical symptoms should be ascribed to the atrophied condition of the liver and diseased state of the kidneys rather than to the disease of the veins. The formation of the obstruction in the veins, it is justly remarked, was most probably due to an attack of uterine disease, but of which there is no history. There could be little doubt that the course of the venous circulation had been entirely re-established before death, the largest portion of the blood, probably, having found its way into the superior cava, through the vena azygos.

"That vessel was found greatly dilated, being fully double its ordinary size, and the intercostal branches, and those proceeding from the spinal canal, were also much enlarged. The freedom of anastomosis between both divisions of the vena azygos, and the spinal and lumbar veins, would afford a ready channel for the returning column of blood, which may have reached those vessels through the medium of the ileo-lumbar and circumflexa ilii veins. The communication between the left emulgent vein and the commencing branch of the vena azygos minor would allow the return of the blood from that vessel.

"Though the branches of the vena porta freely anastomose with the systemic veins, more especially through the medium of the vena mesenterica inferior, and may thus, in cases when the cava is obstructed below the entrance of the hepatic veins, assist in maintaining the circulation, it is not probable that in the present instance this channel was materially concerned in conveying the blood from the lower extremities to the heart. The liver, it will be seen, though free from other morbid change, was very much reduced in size, being less than half its usual weight; the portal circulation must therefore have been much impeded, and hence, most probably, the dilatation of the venous branches on the surface of the liver. Mr. Kiernan has shown that the capsular veins of that organ are branches of the portal system, and anastomose with the phrenic veins, and thus become the medium of a collateral circulation, in some cases of atrophy of the liver. M. Reynaud, in a case of obstruction of the vena cava and portal trunk, found the veins on the surface of the liver dilated, and traced them to a common trunk which pierced the diaphragm, and opened into the cava above the seat of the obstruction." P. 9.

The author sees no reason to suppose that the integumental veins of the abdomen and thorax, and the epigastric, thoracic and mammary veins materially contributed to form collateral channels for the conveyance of the blood.

The disease is viewed by Dr. Peacock as evidently inflammatory in its origin, though the history is too defective to enable him to assign the period at which the inflammation had occurred. From the same cause it is impossible to ascertain the relation which the disease of the veins bore to the affection of the kidneys, which was the immediate cause of death.

This case, though imperfect, both in respect to its history, and the post mortem examination (for which, however, we attribute no blame to the author), is of so rare a nature, that it is perhaps deserving of record in the volume before us. Dr. Peacock has increased the value of the paper, by adding an Appendix, containing references to the cases of obstruction of the superior and inferior cava previously published. He classes them under the four following heads. 1. Obstructions resulting from inflammation of the coats of the veins. 2. Obstructions resulting from pressure on the trunks of the veins. 3. Accumulation of morbid deposits in the cavity of the veins. 4. Obstruction of the veins by coagula resulting from stasis of the contained blood. This Appendix includes a large number of cases, of many of which a brief outline is given. It evinces considerable research, and will be useful to others desirous of investigating the subject.

II. On the Classification, Structure and Development of the Echinococcus Hominis, showing reasons for regarding it as a Species of Cysticercus. By *Erasmus Wilson*, F.R.S. &c.

The author's observations have been limited to two cases of hydatids, instances of acephalo-cyst of the liver, in both of which he discovered the animalcule in question. He was led by this circumstance to infer that, the echinococcus must be more common in its occurrence than had previously been imagined, and he found his opinion corroborated by that of Mr. Curling, who, in a note to Mr. Wilson, states that this, instead of being as he once supposed, a rare form of hydatid, is the most common one infesting man. Dr. Livois, in a work lately published in Paris, entitled "Recherches sur les Echinococques," considers them to exist in every case of acephalo-cyst. From these statements, the author remarks, it is obvious that the echinococcus is a common parasite of the human frame, and that it is probably present in the majority of instances in which acephalo-cysts occur. The term echinococcus was applied by Rudolphi to the acephalo-cyst and its contained animalcules in conjunction. Müller employs the term in its more significant meaning, as referrible to the animalcules alone. The former use of the term assumes a speciality in the acephalo-cyst, while the latter recognises the animalcules as independent beings, or as parasites of the common acephalo-cyst. Mr. Wilson's observations lead him to the latter conclusion, and they further go to prove that the echinococcus has no claim to consideration as a distinct genus, but is in reality a species of the genus cysticercus, to which species, for reasons hereafter to be detailed, he thinks the term "*cysticercus pedunculatus*" peculiarly applicable.

"The acephalo-cyst in which the echinococcus is found, is identical with that in which the animalcules are absent. Nothing is more true than the fact stated by Müller and admitted by Dr. Livois, that in some acephalo-cysts the animalcules are present, while in others, from the same subject, they are absent, and there may be cases in which they are wanting in all the cysts, although such cases are probably rare. The acephalo-cyst is composed, as is well known, of two tunics—an outer tunic, which is semi-transparent, laminated, finely granular in minute texture and highly elastic; and an internal layer, consisting of a thin and very delicate membrane studded with innumerable transparent cells, varying in extremes of measurement from $\frac{1}{10000}$ to $\frac{1}{300}$ of an inch in diameter; but having a medium size of $\frac{1}{2000}$ of an inch.

"This membrane is the seat of development of the echinococcus, and to this, in a fresh acephalo-cyst, they are found connected by means of a very delicate proper membrane, either singly or more commonly in clusters, varying in numbers of individuals from two to one hundred. Some idea of the numbers of echinococci inhabiting a single acephalo-cyst of moderate size, may be inferred from the fact, that in one cyst of the size of a small hazel-nut I counted forty clusters; in several of the clusters there were eighty individuals, and in the entire cyst not less than one thousand. Now, when it is recollected that the majority of acephalo-cysts in an ordinary hydatid tumour would be larger than the one here described, and that of these there may be from one hundred to one thousand present, the entire number of living beings nourished at the expense of the fluids of the diseased person, in such a case, must be enormous." P. 24.

We shall not follow the author in the minute description which he has given of the structure and development of the echinococcus, as few of our readers would understand it without reference to the figures in the plate by which the paper is illustrated. The animalcules have been accurately described by previous observers, and the only novelty that we perceive in Mr. Wilson's paper is an account of the *peduncle,* by which the animal is fixed, either to its own proper membraue, or to the internal membrane of the acephalo-cyst,

"The peduncle is cylindrical, short and granular. When the animal is separated from its attachment, the peduncle offers a variety of appearances, depending on the manner in which it may be torn, and when it is broken close off, an opening is left, which communicates with the internal cavity of the body of the animal. Through this opening I have frequently seen nucleated cells expelled by compression." P. 30.

In proof of the identity of the echinococcus with the cysticercus, the author mentions that all the characters of the latter, viz. a head provided with hooklets, a neck supporting four suctorial processes, and susceptible of retraction within the body, and a membranous cyst-like body, are present in the echinococcus; the situation, the number, the general form of the hooklets, and their arrangement, are the same in both, and the suctorial processes are the same. The peduncle, however, is an important distinction, and merits, Mr. Wilson thinks, the notice bestowed on it in the paper.

This communication is creditable to Mr. Wilson, as a minute and accurate observer, and we may add, that the figures by which it is accompanied are well executed. But as the paper has no practical bearing, and illustrates no point in pathology, we consider it more suitable for the pages of a zoological journal than for the Transactions of a Medical Society.

III. A Case of Aneurism of the Popliteal Artery, cured by Compression of the Femoral Artery. By *Edward Greatrex*, Esq., Surgeon, and *W. T. C. Robinson*, Esq., Assistant-Surgeon of the Coldstream Guards.

On the 2nd May, 1844, Private John Hedley, æt. 27, walked to the hospital, and complained of pain and swelling behind the right knee, which had obliged him to fall out of the ranks on returning from a quick drill in Hyde Park. "On examination, a large irregularly-shaped aneurism was found filling up the popliteal space, strongly pulsating, and admitting of being partially emptied by pressure. It was tender and painful, the leg and foot somewhat swollen, and veins turgid. The swelling prevented his straightening the knee-joint, the circumference of which, taken over the middle of the patella, was $17\frac{1}{4}$ inches, but of the sound knee only $15\frac{1}{2}$ inches. He had perceived the tumour only a few days before his admission into the hospital." He had every appearance of strong health. When he had been in the hospital a week he was attacked by acute laryngitis, which required active treatment. On his convalescence, in the beginning of June, it was determined to treat the aneurism by pressure applied to the femoral artery.

"An instrument was made for the purpose by Mr. Weiss. It consisted of an Italian tourniquet, a broad short splint to fit the outer and back part of the thigh being substituted for one of the usual pads, as less likely to cause pain and sloughing.

"It was first applied on the 18th of June, on the artery at about six inches below the fold of the groin, and the pulaation of the aneurism stopped; but on visiting him shortly afterwards it was found to beat again with unabated force, and this was the case over and over again, although the shape of the pad was changed, and various other little alterations tried.

"He had now the ill luck to be attacked by modified small-pox, which, although slight, occasioned necessarily some relaxation in the treatment of the aneurism.

"The same constant escape of the vessel from beneath the pad occurred until the 8th of July, notwithstanding pressure was made as strongly as he could bear it, or as was advisable for fear of sloughing. The knee then measured 18¾ inches, and the tumour had become softer on the outer side, the pulsation and bellows-sound being very strong and loud. On the 8th of July the plan was adopted of screwing the pad down firmly, and leaving him the key, so that he might, when the pain became intolerable, and 'burning like a hot iron,' to use his own words, relax the pressure by turning the screw for an instant or two, taking care always to apply his thumb or fingers with all his force on the artery just above, in order that during these short but frequent intervals the passage of the blood should still be retarded, if not stopped.

"On the 9th of July this method was found to have been successful, the tumour was now perfectly solid, and no pulsation or bellows-sound has since been detected in it.

"The instrument was kept applied as described, for nine days after the pulsation had ceased, to avoid any risk of the stream of blood again making its way into the aneurism: on the afternoon of the 18th of July it was entirely removed, Mr. Stanley agreeing in the opinion that all danger had passed. The skin under the pad was slightly inflamed, and three or four small blisters had risen there; beyond this, there was no damage done by the instrument. The superficial femoral artery was clearly felt to pulsate down to its entrance into the tendinous canal, though perhaps not quite so forcibly as in the opposite thigh. Two arteries as large as crow-quills employed in the collateral circulalation, were felt passing downwards on the surface of the now hard and solid tumour.

"On the 9th of August, the tumour having diminished, he was allowed to walk about on crutches. On the 24th he walked well with a stick, and could put his heel to the ground. From this time the progress of the case may be told in a few words. He has been kept in the Hospital, but allowed to be up and about all day. Absorption of the contents of the aneurism has proceeded very slowly; with a view of hastening it, friction with stimulating liniments, and gentle pressure by the application of a flannel bandage, have been used.

"On the 8th of November the measurements were, of the right knee 16¾ inches, of the other 15½ inches: although he can perfectly straighten the joint, he finds it easier to walk with it slightly bent. The tumour in the ham is hard, flattened, and yet nearly as large as a hen's egg; on its surface the vessels abovementioned are still perceptible. His general health has been very good, and he has grown fat and stout. On the 14th November he was dismissed the Hospital, and returned to his battalion in the Tower, to undertake light duty." P. 43.

He subsequently resumed full duty, and on the 6th of June, 1845, was stout and well.

This case is a successful example of the treatment of aneurism by a method which has lately been revived, and carried out with more efficient instruments, and in a better manner, than formerly. In the old plans of attempting the obliteration of the aneurismal sac by pressure on the main artery of the limb, the force was so applied as to interfere with the collateral circulation, as well as with that of the vessel leading to the aneurism, and the consequence was, that the plan was not only extremely painful, but attended with greater risk of producing mortification, than the placing of a ligature on the vessel, and the numerous failures, and even fatal results which ensued, led to its abandonment. In the mode in which pressure has recently been applied, the collateral channels have not been interfered with. Within the last few years, some surgeons of eminence in Dublin are reported to have practised compression with considerable success, and it has likewise been tried in several of the hospitals of this metropolis. The subject, however, of this method of treating aneurism of the great vessels of the extremities, including the mechanical apparatus for its application, the amount and duration of the pressure, the cases best suited for this plan of cure, and the grounds for its preference to ligature on the artery, is of too much importance to be dismissed in our brief notice of Mr. Greatrex's case. We can only remark that, in cases of popliteal aneurism of moderate size, in which the surrounding parts have not been much disturbed, the patient being young and healthy—in fact, in such a case as the one here detailed, we strongly recommend the trial of this plan of treatment.

### IV. On Extravasation of Blood into the Cavity of the Arachnoid, and on the Formation of the False Membrane which sometimes envelopes these Extravasations. By *Prescott Hewett*, Esq.

The extravasations of blood treated of in this paper have of late years been carefully examined by several French pathologists, as one of the forms of their "Apoplexie des Méninges;" but these affections have not, as far as the author is aware, received from English pathologists that degree of attention which they will be found to deserve. Blood extravasated into the cavity of the arachnoid may there be found in different states, which, for the sake of perspicuity, the author arranges in four divisions, according to their degree of simplicity.

"1. The extravasated blood may be either liquid or coagulated; if in the latter state, it may be in clots, or spread out in the shape of a thin membranous layer, covering a greater or lesser extent of the surface of the brain.

"2. Sometimes the extravasation presents itself under the shape of a false membrane, possessing more or less of the original colour of the blood; in some cases it is even reduced to the fibrine only, and of a slightly yellowish tinge.

"3. The blood may be fixed to the free surface of the arachnoid, and there maintained by a membrane, which to the naked eye presents all the characters of the serous membrane itself.

"4. The blood is frequently found enclosed in a complete cyst, of various degrees of thickness, which may be removed unbroken from the cavity of the serous membrane.

"The four divisions above referred to may be, and often are, combined with each other, but, in whatever state the extravasated blood has been found, it has, in the majority of cases, corresponded to the upper surface of the brain, and has been rarely met with in the cerebellic fossæ." P. 46.

The author observes that the main feature of the first division is, that it forms a most important link in the appearances about to be examined. He gives, as an example, the case of a patient who died of phthisis, having been repeatedly attacked with delirium shortly before death. "Covering the free surface of the parietal arachnoid, which lines the right middle fossa of the skull, was a large coagulum of blood, measuring, in some places, two lines in thickness. This coagulum, of a fawn colour towards its cerebral surface, was flattened and spread out, but it had not contracted any adhesions with either of the layers of the arachnoid. The brain and its membranes did not present any other trace of disease; the source of the hæmorrhage was not discovered."

The thin membrane-like layer of coagulated blood just mentioned, evidently gives rise to the appearances noticed in the second division. Once thus spread out, the coagulum, after some little time, loses its colouring matter; the fibrine, of different hues, is left, and then gives rise to a false membrane of various degrees of thickness.

After detailing a case, which affords a good illustration of these appearances, Mr. Hewett remarks :—

"The false membranes, arising from extravasation of blood, may be found loose in the cavity of the arachnoid, attached to one of the free surfaces of that membrane; or they may become a bond of union between its two layers, and this, too, without the intervention of any inflammatory action.

"In the great majority of cases, however, the false membrane is connected with the parietal arachnoid only, to which it is pretty firmly attached; its free surface corresponding to the visceral arachnoid, being perfectly smooth and polished, and presenting all the characters of a serous tissue; under these circumstances it is generally supplied plenteously with blood-vessels. When thick, this membrane is often-times divisible into distinct layers; of which, the external ones are of a light colour, whilst the internal ones still possess more or less of the colour of the blood. Clots of blood, of various sizes and colours, are not unfrequently found, either in the structure of the membrane, or upon one of its surfaces. These clots may have proceeded either from the vessels of the arachnoid, or from those of the adventitious tissue. Cysts containing serum and blood, or serum alone, are also met with in these membranes.

"The newly-formed tissue is at times soft and pulpy; at other times having existed for a long period, and undergone various changes, it presents a fibrous, a leathery, and even a cartilaginous appearance. In these states, if firmly attached to the parietal arachnoid, the membrane is very likely to be mistaken for diseases of a very different character; thus, the appearances now under consideration have been referred to chronic inflammation, either of the dura mater, or of the arachnoid, with thickening of these membranes; they have also been described as the result of inflammation of the meninges, producing an exudation of coagulable lymph upon the free surface of the arachnoid." P. 52.

Mr. Hewett refers to 13 cases minutely described by Bayle, under the head of "chronic meningitis with false membranes in the cavity of the arachnoid," in some of which, if not in all, he thinks the membrane owed its origin to an extravasation of blood, and not to an exudation of lymph the result of inflammation, as that author supposes.

After stating his reasons for this, which we are inclined to believe, with him, is the correct view of these cases, he adds that "Mr. Baillarger, who has had frequent opportunities of examining these membranes, under circumstances precisely similar to those of Dr. Bayle, has also come to the conclusion that, in the majority of these cases, the membranes are the result of an extravasation of blood, of more or less standing."

In the third division are classed the cases in which the extravasated blood is fixed to the free surface of the parietal arachnoid by a fine delicate transparent membrane, apparently possessing all the characters of the serous membrane itself, of which it, at first sight, appears to be a part. Two cases, being good examples of these appearances, are detailed by the author, who observes, that this class of cases derives an increased interest from the fact, that these extravasations of blood were for many years described as having taken place between the dura mater and the parietal arachnoid, which membranes were thus supposed to have been widely separated from each other.

"The pathological investigations carried on within the last few years by Messrs. Longet, Baillarger, Calmeil, Ernest Boudet, and others, have all shown that the fine delicate membrane which covers these extravasations of blood, and which presents to the naked eye all the characters of a serous tissue, is a newly-formed membrane, so beautifully adapted to the original serous membrane, that it is only with the utmost care that the exact limits of each can be defined. The cases of this nature which I have examined, have afforded me an opportunity of verifying the accuracy of this opinion; and in speaking of the formation of this membrane, I shall bring forward several examples, which will, I trust, prove that it is much more frequently and much more rapidly formed than is usually supposed.

"Blood extravasated, and thus connected to the parietal arachnoid, may present itself under two different aspects: it may either be collected in one cavity; or it may be disseminated in patches, of various sizes and thickness, over the surface of the serous membrane, the intervening parts of which present a natural aspect." P. 59.

To this class of cases Mr. Hewett is "inclined to refer the patches, of various sizes, of a reddish brown or dark yellow colour, described by Dr. Foville as separating the arachnoid from the dura mater; with which patches this author, in another paragraph, states that extravasations of blood are not unfrequently connected. The patches are, I presume, nothing but these disseminated extravasations of blood, which have lost more or less of their colouring matter. Even Dr. Foville, although he classes these appearances under his 'Meningite parietale,' states that their inflammatory nature is not sufficiently well established for him to enter into a detailed account of the various circumstances connected with them. In this class of cases the collections of blood have never, that I am aware of, been found fixed to the free surface of the visceral arachnoid; even where the blood has proceeded from a laceration of the brain, I have found it fixed to the parietal layer of the serous membrane."

The true nature and situation of those collections of blood, described by many pathologists as being situated between the dura mater and its arachnoid lining, are most easily detected in the cases classed in the fourth division, when the blood is contained in a completely closed bag, which may be detached from the parietal arachnoid, and, with its contents, removed unbroken from its situation. The author remarks, that the existence of these encysted collections has been known to pathologists for many years, but their identity with the supposed extravasations of blood between the dura mater and the arachnoid was, as far as he knows, first pointed out by Mr. Baillarger in 1834. The appearances which these collections of blood present, are well exemplified in the following case. E. W., æt. 51, admitted into St. George's Hospital with paralysis of the limbs and nearly in a state of insensibility, had typhoid symptoms, and died. For the last two years he had been complaining of his head. On examination of the body, Mr. Hewett "found the frontal bone studded, in several places, with deposits of a scrofulous nature, which had, apparently, been originally situated in the diploë, from whence they had, in some parts, caused the absorption of both tables, and in other parts that of the external table only. The external surface of the dura mater presented no morbid appearances worth noticing. Connected with the parietal arachnoid were two remarkable collections of blood, one on either side of the falx. At first sight these extravasations appeared to have taken place between the dura mater and its serous lining, dissecting off the latter to a great extent; but, after a careful dissection, it was ascertained that these appearances depended upon a false membrane, which formed over each extravasation a complete cyst, connected by one of its surfaces to the parietal arachnoid; whilst the other surface, being perfectly smooth and polished, presented all the characters of the original serous tissue, to which it was so beautifully adapted, that it was with difficulty ascertained where the respective membranes began. The connections of the left cyst with the serous membrane were somewhat looser than those of the right, so that that cyst was more readily peeled off from the arachnoid, which was found somewhat roughened, but not discoloured. Each of the false membranes extended far beyond the cysts, and lined the greater part of the parietal arachnoid, corresponding to the upper surfaces of the hemispheres; but the right cyst was much larger, and much more prominent than the left. The walls of the cysts were much thicker than any other part of the false membranes, which were bevelled off, and gradually lost upon the serous tissue; on the left side, the cyst was about a line in thickness. The right membrane was of a yellowish brown colour; the left was of a light yellow. The cavity of the right cyst contained bloody fluid and coagulated blood, amounting in all to about ℥iv.; the coagula presented various colours—some were dark, some of a rusty, and others of a yellow-ochre colour; the cavity of the left cyst was not more than a quarter of the size of that of the right, and its coagula were much more solid. Both cysts were perfectly smooth on their internal surfaces, except at a few points, where some fibrinous coagula were adhering to them. The margins of the membrane on the right side presented, at their junction to the serous tissue, a network of vessels, most beautifully injected, which proceeded in countless numbers towards the cyst. The visceral arachnoid was throughout perfectly sound, and unconnected with either cyst; but on the right side, both the brain and its investing membranes were deeply tinged with yellow. The hemisphere of the brain, corresponding to the right cyst, presented a deep cup-like surface. The substance of the brain, with the exception of the discolouration above mentioned, was healthy in its aspect, but its ventricles were distended with serum. The superior longitudinal sinus, and the upper part of both lateral sinuses, were perfectly healthy. The source of the hæmorrhage remained undiscovered. There were no marks of external violence about the skull."

The author proceeds to remark:—

"Encysted collections of blood, such as those described in the preceding case, have been found in various parts of the cavity of the arachnoid; but in the great majority of cases, these collections correspond to the upper surface of the hemispheres of the brain. The connections of these bags with the free surfaces of the serous membrane in which they are lodged, are sometimes so slender that they appear all but loose; at other times, these bags are pretty firmly connected both with the parietal and with the visceral arachnoid, but most frequently they are connected with the parietal arachnoid only, to which they are sometimes most beautifully adapted; and, in this case, so much do they, on their free surface, partake of the character of an original serous tissue, that they appear, at first sight, to be a part of diseased and thickened arachnoid stripped off the fibrous membrane.

"If the disease be of some standing, the cavity of the cyst may be perfectly smooth and polished, or it may be intersected by fibro-cellular bands, running in various directions; sometimes fibrinous clots are found adhering to this internal surface.

"After a certain period, the membranes forming these cysts become thoroughly supplied with blood-vessels, which may be seen in countless numbers, permeating their whole structure; in several cases it has been noticed, that a minute network of vessels, most beautifully injected, is found at the margins of the new tissue, thus marking the point of union between the original and the false membranes. Thus organised, these cysts possess all the physiological characters of an original serous membrane; they secrete, they absorb; they have been found filled with clots of fibrine and blood-tinged serum; sometimes they contain serum alone, of various colours, and oftentimes, in the cavity of the same cyst, are found coagula of blood of various hues, some recently effused, and others of long standing. In one case, given by Abercrombie, all the contents of the cyst had disappeared, and the cyst itself was found flattened and perfectly collapsed." P. 66.

The latter part of the paper, from which we have quoted rather largely, is certainly the most original; as the author justly states, in the history of these encysted extravasations in the cavity of the arachnoid, one of the most interesting studies is that of the formation of the false membrane by which the extravasated blood is subsequently, either partially or wholly, surrounded. The most generally received opinion is, that an exudation of lymph, consequent upon the arachnoid being irritated by the presence of blood, takes place, and surrounds the extravasation; this explanation, it is remarked, may serve for some few cases, but it will certainly not do so for the majority. The author is convinced that the opinion, broached by some pathologists, of the membranes being, *in some cases*, formed from the blood itself, will be applicable to *a very large majority of these extravasations*. He brings forward several examples to prove that this membrane is wholly formed by the coagulated fibrine of the extravasated blood, and not by a secretion of lymph, as is usually supposed. These examples consist of cases of *blood extravasated into various serous cavities, and covered over by a false membrane, without the least trace of inflammation in the neighbouring tissues*. Six cases are detailed to establish this point, and having, as he supposes, thus proved that a membrane presenting the characters of a serous tissue, is formed from blood extravasated, the author adduces some cases of a newly-formed membrane being produced from the blood in the vessels themselves, and that, too, without any apparent signs of inflammation. The production of this membrane may take place either on the coats of the vessels or on the surfaces of clots contained in the vessels. In a case of an aneurismal pouch situated above the aortic valves, it is stated, "its internal surface was lined throughout by a fine transparent membrane, perfectly smooth and polished on its free surface, except at a few points where some small fibrinous coagula were adhering to it. This membrane and the lining membrane of the artery were, throughout the whole circumference of the aneurismal opening, perfectly continuous, and so beautifully adapted to each other, that, to the naked eye, they appeared to be identically of the same character. In the preceding case it appears to me that the internal and middle coats of the artery had been destroyed, and that the pouch was formed by the cellular coat lined with a membrane which owed its origin to the fibrine of the blood constantly circulating through this abnormal cavity, and which had subsequently taken on the characters belonging to the serous coat of this vessel."

On examining the body of a man who died of diffuse cellular inflammation of the inferior extremity terminating in gangrene, in the superficial and common femoral veins, Mr. Hewett "found extensive clots, not completely filling up the veins, but slightly adherent at different points, to their internal coats. These clots still retained in some places the colouring matter of the blood, whilst at others the colourless fibrine alone remained; in both the veins the clots were enveloped in a perfectly distinct, transparent; smooth, and polished membrane, presenting the appearance of a serous tissue. In the structure of these membranes were several distinct arborescent vessels, minutely injected; some of these vessels were of sufficient size to allow of the blood being made, by gentle pressure, to circulate through them; but no communication could be traced between these vessels, and the coats of the veins. The membranes were easily peeled off from the surfaces of the clots with which they were in contact. The internal coats of the veins presented their natural colour, and polished surfaces, except at the points where the slight adhesions above-mentioned existed."

Mr. Bowman, who had an opportunity of examining with the microscope newly-formed membranes similar to those described in the preceding cases, mentioned to the author the curious fact, that he had never yet found any epithelial cells on the smooth polished surfaces of these membranes, although they, to the naked eye, appear to be exactly of the same nature as the membrane which lines the arteries.

The clots in the heart and large vessels of persons who have died a lingering disease, sometimes present perfect types of the manner in which the extravasations of blood into serous cavities become encysted. The upper surface of the clots, in the cavities of the heart, as this organ lies in its natural position, is not unfrequently covered over by a delicate, transparent membrane, of a whitish colour, and perfectly smooth, aad polished on its free surface, which may be easily peeled off from the blood, with which it is in contact. Even blood extravasated into the tissues, becomes enveloped by a cyst, the formation of which may be, in some cases, traced to the fibrine of the extravasated blood. The well-known apoplectic cyst owes, the author thinks, its origin, in many cases, to the fibrine of the blood already extravasated into the substance of the brain, and not, as is commonly supposed, to an exudation of lymph consequent upon irritation and inflammation of the neighbouring parts.

"Extravasations of blood into the cavity of the arachnoid have been found perfectly circumscribed by a false membrane, presenting all the characters of a serous tissue, six days after the first appearance of the symptoms. I have seen an extravasation of blood into the cavity of the pleura thus circumscribed five days after the accident. If the membrane is, as I suppose it to be in the majority of cases, formed by coagulated fibrine, there is no reason why these extravasations should not be found circumscribed, even at an earlier period; for I have already stated that I have repeatedly found the coagula of the large vessels twenty-four hours after death, completely encysted in a membranous layer of coagulated fibrine.

"By whatever process formed, these new membranes, in the cavities of serous tissues or in vessels, are so beautifully adapted to the original serous tissue, that it becomes a matter of the greatest nicety to trace the respective limits of each." P. 79.

Connected with these extravasations, Mr. Hewett alludes to a remarkable circumstance, viz. an intermission in the symptoms either of coma, or even of paralysis. This intermission may be dependent upon an interruption in the pouring out of the blood, during which time the brain gets accustomed to the pressure and recovers its functions, until a fresh extravasation takes place. This intermission also occurs in those cases where the extravasated blood has been completely surrounded by a perfectly organized cyst, which, by pouring out a fresh quantity of blood or serum into its cavity, may produce symptoms of compression, which will vary according to the more or less rapid absorption of the fluid.

This paper possesses considerable pathological interest. It has evidently been prepared with pains and care, and the latter part, relating to the organization of effused blood, contains observations of an original character which cannot fail to increase Mr. Hewett's reputation as a scientific pathologist. An extensive experience in similar researches enable us to bear testimony to the accuracy of his views.

V. On the Colostrum of the Cow. By *John Davy*, M.D. F.R.S.

The Colostrum—the new milk of the cow after calving—differs from ordinary cow's milk, in being of a richer yellow colour, less liquid, of greater specific gravity, and in possessing the property of coagulating when heated. Resembling in the last-mentioned property the serum of the blood, or the substance of the egg, it has been supposed by some pathologists to be more animalized than common milk, and to contain even serum, and that its being coagulable by heat, is owing to the presence of serum. This inference not having appeared to the author to be proved in a satisfactory manner, he was induced to subject it to some trials, the results of which, in his opinion, tend to show that serum does not enter into the composition of the colostrum, and that its coagulability by heat depends on a peculiar modification of its caseous portion.

We must refer those of our readers who may be interested in the experiments which led Dr. Davy to this conclusion, to the paper itself. He remarks:

"Physiologically considered, the most marked circumstances belonging to the colostrum, are the concentration of nutritive matter in it; the greater facility of its coagulation by rennet, compared with older milk, and its greater power of resisting change when exposed to the action of atmospheric air. These are qualities which may be eminently serviceable, viewing it as the first food of the young animal. Its easy coagulation may suit it to the stomach, in which probably the gastric juice at first, is in small quantity and feeble. Its power of resisting change, and remaining semi-fluid, may adapt a part of it to the intestines, to promote the removal of the meconium. Whilst its concentration as nutritive matter may fit it to perform for the calf the same part that the substance of the egg serves which enters the intestine during the latter stage of fœtal development in the instance of birds, reptiles and fishes." P. 91.

Dr. Davy adds :—

"If the special use of the colostrum of the cow is such as I have inferred, it may be expected that the first milk of other animals will be found to be similar in its properties,—at least, of all those the young of which, like the calf, are born fully-formed and vigorous, with good use of their limbs almost immediately after birth. And so far as I have been able to learn, this is so. The new milk of the ewe, of the mare, and of the sow, I am informed by intelligent farmers, is as rich and thick, and, in the instance of the two first, coagulates when heated." P. 92.

Whether the first milk of those animals, the young of which are born helpless and feeble, as of the carnivora, is also like the preceding, cannot, the author states, be at present determined for want of facts. He is disposed, however, to conjecture that it is similar. In the carnivora, it may be requisite for the first milk to be rich, from the manner in which these animals feed, having to leave their young to procure food, and to be absent, it may be, an uncertain time. And, in accordance with this, is the fact, that the human milk, the first drawn, is not unusually rich, and does not coagulate when heated ; at least, these are the results of the few trials of it that Dr. Davy has made. The "peculiar dilute state of the human colostrum, if proved to be general, seems equally suitable to the condition of the offspring and mother, the one helpless and feeble, not requiring concentrated nourishment, the other commonly, from a certain degree of exhaustion during labour, ill fitted to yield such a supply ;—and, moreover, being designed for domestic life and support, and not under the necessity of separating herself from her offspring to go in quest of precarious food ; offering in this point of view, another instance to the vast number of examples of harmonious adaptation ; and it may be also one circumstance more, by which man as an animal is distinguished from every other."

### VI. Case of Obstruction of the Large Intestines, in which the Ascending Colon was opened with success: the Patient dying three months afterwards of another Disease. By *Samuel Evans*, Esq., of Derby.

Lewis Street, æt. 23, a farmer of robust frame, has for several years been liable to attacks of diarrhœa. In September, 1843, he ate a large quantity of sloes, and was afterwards seized with violent symptoms of colic, which were relieved by bleeding and active aperients. In January the attacks of colic recurred. They became more severe on the 5th of February, and Mr. Evans saw him for the first time on the 7th. "He was suffering from severe pains recurring at intervals of five or ten minutes; the abdomen was greatly distended, but free from tenderness on pressure. The right iliac region appeared to stand out in relief from the distended abdomen; the pains were principally felt in this situation, but extended to the right loin, and to all parts of the abdomen: he had not had any evacuation from the bowels since the 5th, notwithstanding the administration of the most active aperients prior to my visit." Notwithstanding the remedies adopted, no relief was obtained. On the 12th the author "found him vomiting large quantities of olive-coloured stercoraceous matter, and suffering from most agonizing pains in the abdomen; he attributed this attack of pain and vomiting to a dose of castor oil, which had been taken a short time previously. One hundred minims of liq. opii sed. were given, which relieved him in half an hour." On the 13th, several small portions of fæcal matter, and a large volume of flatus were brought away by a soap-and-water injection, after which the distension of the abdomen was diminished. From this time to the beginning of April he continued to get worse. It was quite obvious that there existed a partial obstruction in the bowels, since, the distension of the abdomen, and the *swelling in the right iliac region*, had considerably increased. "The patient had become much emaciated, was extremely weak, and his general health greatly impaired; the vomiting recurred almost daily, and the stomach could retain only the smallest quantities of food; the lower extremities were much swollen, the tongue and skin dry, the pulse frequent and small."

On the 25th March, Mr. Evans represented to the sufferer that relief might probably be afforded by the formation of an artificial anus in the loin, but in compliance with the wishes of his friends, the operation was postponed.

April 8th. The patient being much emaciated and in a sinking state, and the abdomen distended to the greatest possible degree, consent was given to the operation.

"The patient having been laid on the bed with his face downwards, two pillows were placed under the belly for the purpose of rendering the right loin more prominent. I made an incision of four inches across the loin, commencing it at the outer margin of the sacro-lumbalis muscle, about an inch above the crest of the ilium, and extending it forwards in a direction parallel to the crest. Having divided the latissimus dorsi, and the outer margin of the quadratus lumborum muscles, I proceeded to expose the bowel by opening the fascia; but in doing so, I also opened the intestine, which was intimately united with the fascia, by dense cellular tissue, without any intervening fat: indeed, no fat whatever was brought into view, as I had been led to expect there would be—a fact that may be accounted for by supposing the extreme distention of the head and ascending portion of the colon, to have pressed upwards the kidney with its circumjacent fat, above the line of incision. Possibly, however, this fat may have been absorbed, as the man was extremely emaciated. The instant the bowel was punctured, a profuse quantity of semi-fluid, clay-coloured fæces was projected with great force through the opening, and continued to escape for a considerable time, so as to amount altogether to more than two gallons. I then enlarged the orifice in the intestine to the extent of one inch in a transverse direction, or parallel to the external incision. The last steps of the operation consisted in connecting the edges of the incision in the intestines, with those of the outer or anterior half of the external wound, by means of five sutures, the inner third of the external wound being closed by a pin, and twisted suture, and covered by a few strips of adhæsive plaster. A small piece of oiled lint was then placed between the edges of the wound in the intestine, and cloths wetted with warm water were laid over the wound. The abdomen, now reduced to its natural size, was supported by a many-tailed bandage, and the patient was placed on his right side in bed, so as to allow of a free escape of the contents of the bowel through the wound." P. 101.

The patient's recovery remained doubtful for a few days after the operation. He subsequently began to improve, and on May 8th, he was gaining flesh; the wound in the intestine was perfectly united to the skin, but the orifice was so reduced in size, as barely to admit a rectum-tube of common dimensions. When this was passed about five inches up the ascending colon, it seemed to meet with an obstacle, and, as it caused pain, it was withdrawn. "As he feels no natural desire to defecate, he finds it necessary to remove the plug from the artificial anus four or five times a-day, whilst he is in the horizontal posture, and the fæces being always semi-fluid, easily escape into a vessel placed under the orifice. Injections of warm water have been administered by the rectum every two or three days, but they have invariably returned mixed with small portions of opaque mucus."

June 8th.—The patient had become quite fat, and there appeared every prospect of his soon regaining his accustomed strength. On the 25th, however, he was attacked with diarrhœa, with symptoms of dyspepsia. On the 28th, a diabetic state of the urine was observed, and on the 2nd of July, after a ride of six miles in an uneasy cart, symptoms of peritoneal inflammation supervened, and he died on the 5th. The body was examined 23 hours after death.

"The artificial anus was a small constricted circular aperture, about $\frac{5}{8}$ths of an inch in diameter; its margin was firm and unyielding; the integuments near the orifice were slightly drawn inwards, and their connection with the coats of the intestine were so intimate, that the line of union of the tissues could not be defined.

"The abdomen contained 6 ozs. of serum, of a dirty yellow colour.

"The stomach and small intestines were greatly distended with flatus; the peritoneal surface of the lower two-thirds of the ilium was highly vascular, and of a florid red colour.

"The mucous membrane of the stomach and small intestines appeared healthy; the latter contained a small quantity of pale yellow chymous matter.

"The cæcum was enormously distended, and was nearly as large as a stomach of ordinary size; the ascending colon was also much enlarged.

"About a quarter of an inch from the commencement of the transverse colon, that is, just beyond the angle formed by the junction of the ascending and transverse portions of the colon, the gut was contracted to about three-quarters of an inch in diameter, and about the same in length; the contracted portion presented a cartilaginous hardness.

"The serous covering of the cæcum, ascending colon, and the first half of the transverse colon, was highly vascular; there were numerous small patches on its surface, of a deep red colour, which were partially covered with recently-effused lymph.

"The anterior three-fifths of the circumference of the first half of the ascending colon were bounded by peritoneum, and the posterior two-fifths by the fascia covering the lumbar muscles; and it was in the latter part of the bowel, about half an inch beyond the cæcum, that the artificial anus was formed.

"About two ounces of dark brown fæcal matter were found in the cæcum and ascending colon; the mucous membrane was of a deep claret colour, and thickly covered with bloody mucus.

"The contracted portion of the colon was almost as hard as cartilage; it formed a ring about three lines thick, and nine lines broad, and appeared to consist of compact white fibres, arranged irregularly around the intestine; between the mucous and muscular coats the stricture would just admit a crow-quill; its inner surface was in a state of ulceration, and presented an irregular granulated appearance without any traces of mucous membrane. The mucous membrane of the first third of the transverse colon was in a state of uniform injection; the whole extent of the tranverse colon was lined with a muco-purulent secretion, of a greenish colour, which was most abundant near the stricture.

"The descending and sigmoid portions of the colon were quite empty and contracted, and presented no morbid appearance." P. 109.

The author remarks—

"This is the eleventh case on record, in which Callisen's operation (modified by Amussat) has been performed in the adult, in consequence of obstruction in the intestinal canal.

"From the previous history of the case, it would appear that the disease had been of slow progress, and of considerable duration. The gradual distention of the abdomen, and the absence of any satisfactory evacuation from the bowels during a period of seventy days, served to give strength to the opinion, that there existed a progressively increasing obstruction in the bowels, which, from the small amount of fæcal matter evacuated during that period, approached very nearly to a case of complete obstruction." P. 110.

The patient was in so alarming condition at the time of the operation, that it was impossible to imagine a case more unfavorable for the operation, but as the patient recovered in health, so as to be enabled in two months afterwards to walk several miles, as far as the operation is concerned, Mr. Evans regards the case as successful. He observes, "if the patient had possessed a sound constitution, there is reason to believe that he might have enjoyed a tolerable share of health, but subject to the inconvenience attending an artificial anus, as the obstruction of the bowels was so complete as to preclude all hope that the continuity of the canal might ultimately be re-established."

This case will be perused with some interest by operating surgeons. The propriety of performing such an operation in a case where some doubt must exist as to the seat and nature of the disease causing the obstruction, has been questioned. We certainly are not likely to meet with many instances in which the indication in respect to the situation of the stricture is so clear as it appears to have been in the above case. The great distension of the colon rendered the operation comparatively an easy one. We have pleasure in acknowledging the skill and judgment displayed by Mr. Evans in the management of the case.

VII. On the Mortality in Prisons and the Diseases most frequently Fatal to Prisoners. By *William Baly,* M.D. Physician to the Millbank Prison and Lecturer on Forensic Medicine at St. Bartholomew's Hospital.

After contrasting the present improved condition of our prisons with that which existed in the time of Howard, Dr. Baly, nevertheless, mentions it as a fact resting on statistical enquiries, that the mortality is much higher among prisoners than among persons of the same age in society at large, and he considers this fact well worthy the consideration of medical men in reference to the causes upon which it may be found to depend.

"The desire," he proceeds to say, "of arriving at a satisfactory conclusion as to the relative activity of different causes of disease in the Penitentiary, led me to institute a rather extensive inquiry respecting the rate of mortality, the nature of the more prevalent fatal diseases and their causes in other penal establishments.

"The main results of this investigation were, first, that imprisonment, continued for periods of 2, 3 or 4 years, produced everywhere a high rate of mortality; and secondly, that, although in particular instances other causes might contribute to increase the number of deaths, yet, in all prisons, the Millbank Penitentiary included, the increased mortality was chiefly due to the prevalence of *one and the same disease*, namely, tubercular scrofula. The facts upon which these conclusions are based, will be detailed in the paper now before the Society." P. 116.

A new epoch in the history of prison discipline in this country has arisen, he conceives, since the prison at Millbank has ceased, in June 1843, to exist as a Penitentiary; since the diet of all gaols has been regulated on a new and improved scale, and new prisons have been erected, to which all the resources of modern science have been applied.

Dr. Baly proposes in the present paper to review only the results of imprisonment during the period of fifteen or twenty years just past, and the plan which he intends to follow is, first, to determine the rate of mortality in the Millbank Penitentiary, and in other prisons, as compared with that of the free population in the same country. Secondly, to point out causes, independent of diet and discipline, which influence mortality in prisons; and lastly, to show the relative prevalence of different classes of fatal diseases amongst prisoners, and demonstrate the influence of duration of imprisonment, diet, season of the year, age, sex, and other causes.

### I. RATE OF MORTALITY IN PRISONS.

### *Rate of Mortality in Millbank Penitentiary.*

A difficulty occurs at the outset in estimating fairly the mortality during the period of 18 years, viz. from 1825 to 1842 (both included), over which the observations extend, for, though the number that actually died in the establishment was 205, giving an average annual per centage mortality of 2·14 upon the average number of prisoners 532 for this period, there were 355 invalided or pardoned on medical grounds, a portion of whom would have swelled the mortality had they not been removed. Dr. Baly, after a careful consideration of all the facts bearing on the subject, considers that 123 of these would probably have died before the expiration of their terms of imprisonment, and that that number must, therefore, be added to the other deaths, making the total amount 328, and the annual per centage mortality 3·42, a rate more than double that of 1·539, *i. e.* the mortality per cent. of the inhabitants of the metropolis between

15 and 70 years of age for the year 1841. But it is to be remembered that this was a healthy year, and that Asiatic Cholera caused 31 of the deaths among the prisoners, which being subtracted, reduce the Millbank mortality to 3·0976, which is still excessive.

After stating the sources of his information, which is gathered over a very wide field, and giving a table which must have cost great labour in its construction, and which is highly interesting and valuable for the results which it displays, the author adds the following summary of what has just gone before.

"It will be seen that the annual rate of mortality ranges in the different prisons in England from 15·767 per thousand to 38·938 per thousand; in the prisons of the United States, from 19·019 to 39·336 per thousand; and in Switzerland from 26·380 to 38·690 per thousand; while in France the rate of mortality of the Hulks varies from 30·7 to 53·4 per thousand, and that of the Houses of Correction from 30·5 to 86·9 per thousand." P. 127.

A table of the rates of mortality between 15 and 70 years of age for London, Paris and other parts of France, for Geneva, and for New York, shows the much smaller mortality in all these places observed among the population at large.

### II. CAUSES INFLUENCING THE RATE OF MORTALITY IN PRISONS.

1. *Pardons on medical grounds* it is shown are granted to those confined in the other English prisons as well as to prisoners at Millbank; and the same practice obtains in the United States also. It is calculated that, on this account, the apparent annual mortality 19·013 per thousand in thirty-six principal gaols and houses of correction in England should be raised to 22·788 as probably nearer the truth. As pardons on account of ill health are not granted to convicts in the Hulks, the mortality is comparatively very high, viz. 38·938 per thousand.

2. *Differences of the Prisoners in respect of race, class, and consequent predisposition to disease.*—Under this head we have M. Villermé's authority quoted for the fact, that the mortality in the different prisons in the Department of the Seine increased in a direct ratio with the wretchedness and poverty of the prisoners, ranging from 1 in 40·88 of those in good circumstances to 1 in 3·97 among the utterly destitute. In the United States, too, it appears that the mortality in prisons is greatly increased where a large proportion of the coloured population is mixed with the whites. The mortality of the white races we find it stated is raised by imprisonment from 13 or 15 to 20 per thousand annually, but that of the coloured race, which in the free condition ranges from 23 to 27, has risen in one Penitentiary during the last 14 years as high as 70 per thousand, and even higher in another prison during the last three years. The high rate of mortality in the English Hulks may be in part referred to the former practice of removing the healthy and able-bodied convicts and leaving such as appeared, from disease or infirmity, unfit for transportation to a penal settlement.

3. *Length of the Terms of Confinement.*—The average term of imprisonment varies greatly in different prisons, and, as a general rule, the rate of mortality is highest in those places where the terms of imprisonment are longest; the Hulks of England, however, the Penitentiary of Lausanne, and the French Maisons Centrale, present exceptions on the side of excess, that is, have a greater mortality than corresponds with the duration of imprisonment. Amongst the American prisons, the lowest rate of mortality is coincident (in the Auburn State Prison) with the longest average term of confinement, while the highest co-exists (in the Eastern Penitentiary) with the shortest average period of imprisonment.

"In consequence of the number of deaths in prisons being liable to increase or diminution from so many different causes, it is impossible to determine the exact influence of the duration of imprisonment, by comparing the rates of mortality in different penal establishments. But we attain this object more satisfactorily when we compare the rates of mortality to which prisoners are subject at different periods of their imprisonment in the same prison."—P. 144.

After explaining the difficulties to be overcome, and the data on which he has proceeded, Dr. Baly thus expresses the result which, in all probability, most nearly approximates to the truth. "The rate of mortality amongst the prisoners in the Millbank Penitentiary, then, appears to have been 13·052 per thousand during the first year of punishment; 35·645 during the second year; 52·267 during the third year; 57·139 during the fourth year; and 44·170 during the fifth year; the rate of mortality rising rapidly from the first to the third year, and slowly in the fourth year; but falling again in the fifth year."—P. 150.

The author closes this division of his subject with the remark, "that in the Millbank Penitentiary, and also in the prisons of France, the mortality has been greater amongst the prisoners who were undergoing their second, third, or fourth year of imprisonment, than amongst those who had been longer in confinement, so that it would seem as if prisoners who were of feeble constitution, or predisposed to disease, generally fell victims to the injurious influences attending imprisonment before the end of the fourth year of their confinement, while those who were able to support their punishment until that period, without serious deterioration of health, were proof against the causes of disease to which they were exposed."—P. 155.

4. *The Locality.*—Dr. Baly points out that the number of deaths occurring in different prisons cannot be taken as any guide as to the relative healthiness of their sites, unless the influence of the other causes already discussed be in the first place ascertained and equalized; except at least in those cases in which the combined effects of those other causes are found equal or nearly so. He conceives that the only sure evidence of the mortality being increased by a noxious influence of the locality, will be the prominent part played by endemic diseases amongst the causes of death; and proceeds to inquire into the nature of the diseases causing death in six of the American prisons. These endemic diseases he considers under three heads, viz.—1. Fevers, exclusive of the exanthemata. 2. Bowel complaints. 3. Hepatic diseases. He shows that, in the Sing Sing State prisons, the mortality of which he has proved to be excessive, to the extent of 6 per 1000 annually, (after allowance has been made for the operation of the other causes already discussed,) the annual mortality per 1000 in the prison, and amongst the population, above 10 years of age, in New York, are respectively from Fever 3·093 and 1·400; from Bowel complaints, 4·860, and ·884; and from Hepatic diseases, ·773 and ·334. The annual mortality per 1000 in this prison, from all these causes together, reaches 8·726, while in Charlestown state prison it reaches only 2·614, though this is the next in amount among the six prisons brought into comparison. The Sing Sing prison, it is remarked, stands on a low flat, at the foot of a hill which completely overlooks it, and close to the bank of a very large tidal river, the Hudson. The conclusion at which the author arrives, that the excess in the number of deaths in this prison is due to the insalubrity of its locality, seems well supported by the facts adduced.

He comes next to discuss the influence of locality in reference to prisons in England; and it results from the table setting forth the mortality in different prisons from the three classes of diseases before-mentioned, that the proportion of deaths caused by them in the principal county-prisons, 32 in number, taken collectively, has been only $\frac{1}{7}$ less, while in some of them it has been greater than in the Penitentiary. The average annual mortality by all the diseases which can be ascribed to the influence of malaria, has been 4·511 per 1000 prisoners in the County Gaols and Houses of Correction, and 5·632 per 1000 in the Millbank Penitentiary; and this excess has been almost entirely due to Fever. In the Wakefield House of Correction the mortality by Fever alone, and that by bowel-complaints alone, has been still greater, and by both together $\frac{4}{5}$ths greater than in the Penitentiary. These results are obtained without any allowance for pardons on account of ill health, which, in reference to these acute diseases, is deemed unnecessary. An examination of the reports of the Registrar-General shows that the mortality from these diseases during recent years has been three times as great in the county prisons of England as amongst the general population of London; this excess being due to that arising from the first two classes of diseases; that from hepatic complaints being less among prisoners than among the community at large. Fever in prisons is far less a scourge than formerly, producing now in ten years not so many deaths as it formerly caused in one.

The author here digresses to consider the influence of the dietary upon the mortality in prisons, after having shown the discordant and contradictory opinions which have been entertained on this question. The injurious effect of a diet, consisting in great measure of liquids, is supported by the fact that, while diarrhœa has prevailed in a prison, the neighbouring population have not suffered in a proportionate degree; and diarrhœa in prisons has followed an impoverished diet, and been stopped or mitigated after its improvement. On the other hand, it may be urged that, in some instances, as in the Salford and Preston Houses of Correction, diarrhœa has been infrequent, although for several years their inmates were allowed less solid meat and bread than in other prisons where diarrhœa prevailed: it is argued, too, that a liquid diet, though it might produce looseness of bowels, could hardly give rise to acute inflammation of the large intestines or dysentery. Improved diet, moreover, has only mitigated, and not removed the evil; and the officers of prisons and their families have generally not escaped. The prevalence of diarrhœa has varied according to the season and state of the atmosphere, and has been greatest when the disease has most raged among the population at large, as in the year 1842. Dr. Baly states, besides, that he has visited many prisons in different parts of England, and has met with none in which the prevalence of bowel-complaints was remarkable, and the site, at the same time, free from obvious sources of malaria. In barracks, the production of diarrhœa by poorness of diet is out of the question, yet some, as the Tower of London, have been distinguished for its prevalence, attributable, probably, to the muddy moat then existing. The conclusion arrived at is, that an enfeebling diet renders the system more susceptible of the influences of an atmosphere slightly impregnated with malaria, which last is considered the general exciting cause of the malady, while the better nourished suffer less, or escape altogether; as happens constantly in aguish districts. The same remark applies in reference to the causes of Fever in prisons.

It is shown, by the result of an experiment on a large scale at the Millbank Penitentiary, that neither the fever nor the bowel-complaints are due to the water in common use. Some obvious practical inferences, regarding the choice of a site, the necessity of drainage, and the improvement of diet in particular cases, conclude this digression. The author next proceeds to show that the large excess of mortality in some English prisons above that of the average amount in 32 county prisons, is only in part accounted for by the increased number of deaths from the endemic diseases before spoken of, and it is the deaths by these diseases that mark the influence of the locality.

"The average annual mortality per thousand prisoners from all causes, in the Penitentiary, (making the addition previously explained for the pardons on medical grounds,) has exceeded, by more than twelve (12·368) deaths, the total average annual mortality of thirty-two county prisons, calculated only from the deaths actually occurring in those prisons. And the deaths caused by fevers and bowel complaints will account for only 2·532 out of that annual excess of twelve deaths. On the other hand, the total annual mortality in the Wakefield House of Correction (without any allowance for pardons on medical grounds) has exceeded the average annual mortality of the thirty-two other county prisons, by rather more than eight (8·135) deaths per thousand prisoners, and of this excess 4·498 deaths per thousand prisoners, or more than half, have been due to fevers and bowel complaints." P. 185.

Having already compared the mortality of prisoners with that of the population at large between the same limits of age, and found the former to be excessive, he proceeds to compare the rates of mortality of the criminal class in and out of prison.

The very great mortality in prisons amounting, when fairly estimated, to 30·976 per thousand annually in the Millbank Penitentiary, and to 22·788 in the county prisons; as well as the established fact of the large increase of mortality among prisoners confined for some years, and the few deaths occurring during the first six months after admission, support the opinion, that the rate of mortality is augmented by imprisonment, and that it is not due to the class from which the prisoners are principally taken. Statistical results obtained in some districts remarkable for poverty and wretchedness confirm this inference; which is shown in all probability to be applicable in America as well as in England. One cause mentioned by the author, as having undoubtedly been highly influential in preserving the health of prisoners in American penitentiaries, is their abundant and nutritious food. As regards America, the conclusion is, that imprisonment raises the annual rate of mortality per thousand from 15· to 20· among the whites, from 27·225 to the enormous rate of 70·10 among the coloured people, and it scarcely admits of doubt that like effects arise in France and Switzerland.

### III. DISEASES BY WHICH THE MORTALITY OF PRISONS IS CHIEFLY PRODUCED.

*Fatal Diseases of the Prisoners in Millbank Penitentiary.*

It has been shown before that the total excess of mortality from fevers, bowel complaints, and hepatic diseases in the Penitentiary, as compared with the metropolis, has been 4·682 per 1000 annually. The mortality from all causes in the Penitentiary has, however, been estimated at 30·976 per 1000 annually, while, in the Metropolis, that for all persons between the ages of 15 and 70, has been only 14·707, thus leaving an excess of 16·269 deaths on the side of the Penitentiary, of which fevers and bowel complaints have produced 4·682, or little more than one-fourth. There remain, therefore 11·587 deaths annually per 1000 prisoners to be accounted for. Now, it appears from a table formed to show the annual mortality from different diseases (exclusive of the three heretofore discussed) per 1000 prisoners in the Penitentiary, and the same number of persons between 15 and 70 years of age in the Metropolis, that the excess in the prison mortality under the classes of Epidemic Diseases, not including fever, &c., Diseases of the head and nervous system, abdominal Diseases, not including fluxes, and Diseases of undefined nature amounting in the aggregate to 2·332 per 1000 annually, is almost exactly balanced by the excess on the side of metropolitan mortality under the classes of Diseases of the heart, Diseases of the respiratory organs, exclusive of Consumption, Diseases of the genito-urinary organs, Diseases not classified, and violent deaths, amounting in the aggregate to 2·352. We have then remaining the two classes, viz. Consumption and other tubercular Diseases, which show an excess of prison mortality of 8·870 and 2·836 respectively, that is of 11·706 together, to account for the before unexplained excess.

This near approximation of numbers is striking; Dr. Baly proceeds to explain the plan on which he has arrived at it, in this our space does not allow us to follow him, we can only say that his assumptions and reasonings appear to us fair and satisfactory.

He then sums up thus:

"Without any exaggeration then of the facts, it seems to me to be clearly proved by the medical records of the Penitentiary, that the mortality caused by tubercular disease has been between three and four times as great during the eighteen years, 1825 to 1842, among the convicts confined in this prison, as it was in the year 1842 amongst persons of the same period of life in London generally; and that three-fourths of the excess of deaths from all causes in the

Penitentiary above the rate of mortality of all persons in the metropolis of the same period of life, has been due to the prevalence of this disease; fevers and bowel-complaints having produced only one-fourth of that excess of deaths." P. 203.

It is subsequently shown that the proportion of persons affected with phthisis among the criminals when first received, has been far too small to account for any considerable part of the great mortality which that disease every year produced among the prisoners confined in the Penitentiary.

### *Fatal Diseases in the English County Prisons.*

It appears, from the tables given, that, though consumption and other forms of scrofula have been more fatal to the prisoners confined in the county gaols and houses of correction than to the inhabitants of the metropolis, they have produced much less mortality in those prisons than in the Millbank Penitentiary. Yet this result does not militate against the belief, that the tubercular cachexia, most frequently fatal in the form of pulmonary phthisis, is the disease which imprisonment has an especial tendency to produce. Even the mortality observed in these prisons from this cause is high, when it is taken into account that the average duration of imprisonment is less than two months. No inference on this subject can be drawn from the case of the convicts in the English hulks, for reasons already adverted to.

As relates to France, some reports published by the physicians of the Maisons de Correction at Nismes and Rennes, show that tubercular consumption has been the principal fatal disease in those prisons. Some confirmation of the same fact is derived from observations made in the Geneva and Lausanne Penitentiaries, though here the results have been obtained only on a small scale.

### *Fatal Diseases in the American Prisons.*

The terms of confinement in the American prisons have been for the most part longer than in the Millbank Penitentiary, the records of these establishments are accurate; but there are no data for determining the increment of deaths due to pardons on account of ill-health; the calculations are therefore made withont any allowance on this ground, a fact which should be borne in mind in drawing inferences from a comparison of the results.

The mortality among the white convicts is first considered in the Eastern Penitentiary, the Auburn and Charlestown State Prisons, and the result arrived at is that the mortality from consumption and hæmoptysis being 4·706 annually per thousand in the male white population of New York, it has been 12·864, 9·526, and 10·787 respectively in these establishments in the order in which they stand. It will be observed, that the greatest excess of mortality from these causes in these prisons above that of the general white population, viz. 8·158, falls short of 11·706, the excess of the Millbank Penitentiary over that of the London population from the same causes. Though allowance is to be made for the pardons not being taken into account, and for the prisoners being of a superior class in the case of the American prisons, our author does not shrink from avowing his belief, that imprisonment in most of the American Penitentiaries has really exerted a less injurious influence on the health than confinement in the Penitentiary of Millbank.

We come next to inquire into the causes of death among criminals belonging to the dark races, and find that the rate of mortality from consumption and other tuberculous diseases being 11 per 1000 annually amongst the coloured male population of the City of New York, is observed to be 40 per 1000 in the Eastern Penitentiary; and even this estimate is probably below the truth. The reports of the Maryland Penitentiary show 28·490 to be the annual rate of mortality from these diseases among criminals of the white and coloured races mixed in nearly equal proportions.

An interesting inference regarding the white convicts is drawn from the experience of the Sing Sing Prison. Both the site of this establishment and the large proportion of deaths from diseases of a notoriously malarious origin, lead us to believe that it suffers pre-eminently from that source of mischief, yet its tubercular mortality of 11·265, is excessive compared with that of New York, which is for whites 5·304, greater than those of the Auburn and Charlestown State Prisons, which are 9·892 and 10·787 respectively, is less than that of the Eastern Penitentiary, viz. 12·864; each of which prisons has suffered in a very slight degree from diseases due to malaria, less than even New York itself; so that we cannot avoid coinciding in the two-fold conclusion of our author, that, as far as our evidence goes, malaria must be considered incapable either of promoting or preventing the development of tubercular disease. This conclusion manifestly weakens the objection to the locality of the Millbank Penitentiary.

Dr. Baly conceives that it has been shown that the increased mortality in the Millbank Penitentiary, has been due almost wholly to the diseases which are characterised by the deposition of tubercular matter in different organs of the body, but principally in the lungs, and that it has further been shown that, in all prisons where convicts have been confined for long terms, the same state of things has prevailed. Farther on he gives some facts more in detail, establishing the belief that other forms of scrofula besides consumption have a tendency to become developed to a more than ordinary extent during confinement in prisons.

He reserves, for a second paper, the investigation of the causes on which this great mortality from tubercular diseases has depended. Among the most influential of which he places, 1st, deficient ventilation; 2nd, cold; 3rd, want of active bodily exercise; 4th, a listless if not dejected state of mind, and 5th, poorness of diet. He concludes with the remark, that the facts which he has detailed do not justify any sweeping condemnation of imprisonment as a system of secondary punishment, but augurs far more favourable results from the measures already in progress for freeing this mode of punishment from most of the injurious conditions which have hitherto generally attended it.

We conclude this long epitome with the offer of our best thanks to Dr. Baly for the very elaborate and able paper which he has brought before the public; its full value can of course only be appreciated by those who study it, yet our sketch is sufficient to show that it must possess the highest authority on the subject of which it treats. We shall hail with pleasure the appearance of his second paper.

